# Development of Magnetic Microwires for Magnetic Sensor Applications

**DOI:** 10.3390/s19214767

**Published:** 2019-11-02

**Authors:** Valentina Zhukova, Paula Corte-Leon, Mihail Ipatov, Juan Maria Blanco, Lorena Gonzalez-Legarreta, Arcady Zhukov

**Affiliations:** 1 Departamento de Física de Materiales, Facultad de Químicas, Universidad del País Vasco/Euskal Herriko Unibersitatea, UPV/EHU, Paseo Manuel de Lardizabal, 3, 20018 San Sebastian, Spain; valentina.zhukova@ehu.es (V.Z.); paula.corte@ehu.eus (P.C.-L.); mihail.ipatov@ehu.es (M.I.); lorena.glegarreta@gmail.com (L.G.-L.); 2 Departamento de Física Aplicada, EIG, Basque Country University, Universidad del País Vasco/Euskal Herriko Unibersitatea, UPV/EHU, 20018 San Sebastian, Spain; juanmaria.blanco@ehu.es; 3 Departamento QUIPRE, Inorganic Chemistry-University of Cantabria, Nanomedice-IDIVAL, Avda. de Los Castros 46, 39005 Santander, Spain; 4 IKERBASQUE, Basque Foundation for Science, 48011 Bilbao, Spain

**Keywords:** magnetic microwires, giant magnetoimpedance, domain wall propagation, magnetoelastic anisotropy, magnetostriction, annealing, internal stresses

## Abstract

Thin magnetic wires can present excellent soft magnetic properties (with coercivities up to 4 A/m), Giant Magneto-impedance effect, GMI, or rectangular hysteresis loops combined with quite fast domain wall, DW, propagation. In this paper we overview the magnetic properties of thin magnetic wires and post-processing allowing optimization of their magnetic properties for magnetic sensor applications. We concluded that the GMI effect, magnetic softness or DW dynamics of microwires can be tailored by controlling the magnetoelastic anisotropy of as-prepared microwires or controlling their internal stresses and domain structure by appropriate thermal treatment.

## 1. Introduction

Magnetic sensors allowing control and monitoring of various processes and functions are essentially relevant for a great number of industries, such as data storage, home entertainment, energy harvesting and conversion, informatics, telecommunications, aircrafts, aerospace, automobiles, electronic surveillance, medicine, biology, etc. over many decades [1,2,3]. 

The essential part of magnetic sensors is appropriate magnetic material. Particularly, soft magnetic materials are used for a wide number of magnetic sensors [3]. 

One of the most prospective families of soft magnetic materials presenting a number of advantages such as excellent magnetic softness, fast and inexpensive fabrication process, dimensionality suitable for various sensors applications and good mechanical properties is the family of amorphous and nanocrystalline materials prepared using rapid melt quenching [4,5,6]. 

Best magnetic softness as well as physical properties linked to soft magnetic properties (i.e., giant magnetoimpedance, GMI) are reported for amorphous and nanocrystalline wires or ribbons [5,6,7,8,9,10]. On the other hand, magnetic wires can also present perfectly rectangular hysteresis loops and single and fast domain wall propagation [11,12,13,14,15,16].

It is worth noting that the observation of both single domain wall propagation and above mentioned GMI are not restricted to amorphous and nanocrystalline wires and were reported in crystalline magnetic wires [17,18]. However, the highest GMI effect as well as the fastest single domain wall propagation are observed in amorphous magnetic materials [19,20].

The main interest in the GMI effect is related to one of the largest among the non-cryogenic effects, impedance sensitivity to an external magnetic field (up to 10%/A/m) reported for magnetic microwires [15,19,20]. Such GMI features reported in amorphous wires allowed development of the GMI sensor technology suitable for various applications, like magnetic field or acceleration sensors integrated in CMOS circuit [21,22], sensitive low-dimensional magnetometers suitable for magnetic field monitoring [23,24], biomagnetic field detection allowing the pico-Tesla sensitivity [25] or magnetoelastic sensors [26,27]. 

The main features of the GMI effect are satisfactorily explained in terms of classical electrodynamics considering the skin effect of a magnetically soft conductor [7,8,9,10]. 

However, like magnetic permeability, GMI effect presents tensor origin [9,10,27,28,29]. Therefore, the domain structure of magnetic materials (domain walls, magnetic anisotropy distribution) can considerably affect the GMI features [9,10,28]. On the other hand, the anti-symmetrical magnetic field dependence of the off-diagonal GMI component observed in amorphous wires is attractive for technical applications [30,31]. 

The miniaturization of the sensors and devices requires employment of thinner soft magnetic wires and hence an extension of the frequency range towards the GHz band [22,23]. In the whole investigated frequency range, the domain walls are strongly damped. Therefore, spin precession and magnetization rotation on the surface layer of magnetic wires must be considered for interpretation of the impedance change upon external magnetic field. Consequently, impedance changes induced by external magnetic field at GHz frequencies have been attributed to the ferromagnetic resonance (FMR) [22].

On the other hand, different families of magnetic wires can present fast and controllable electric current or magnetic field driven domain wall (DW) propagation [13,16,32,33]. These features observed in planar or cylindrical wires are proposed for various applications for data storage (magnetic racetrack memory, MRAM) and magnetic logic devices [33,34]. The peculiarity of the amorphous and nanocrystalline magnetic wires is an extremely fast DW propagation characterized by high DW velocity and mobility reported elsewhere [13,16,32,35,36,37]. The DW dynamics (DW velocity and mobility) can be tuned by the magnetoelastic anisotropy, transverse magnetic field and stresses [35,38].

As recently reported elsewhere [38,39,40], the DW velocity and mobility of amorphous microwires can be further improved by appropriate thermal treatments.

Consequently, from the actual state of art on DW dynamics in magnetic microwires, we can assume that the DW velocity can be tailored by the chemical composition of metallic nucleus as well as by the thermal treatment [33,34,35,36,37,38,39,40]. 

Given the attractive magnetic properties of amorphous wires, the development of magnetic sensors using magnetic wires has aroused great interest in recent years.

Thus, various magnetic sensors employing stability either GMI effect or magnetic bistability of amorphous wires have been developed [21,22,23,24,25,26,27,41,42,43].

Consequently, preparation and processing of thin magnetic wires with improved magnetic properties is essentially relevant for development of magnetic sensors. Therefore, in this paper we will provide the routes allowing optimization of either GMI effect or domain wall dynamics in magnetic microwires.

## 2. Experimental Methods

We studied Fe-, Ni- and Co- based microwires with metallic nucleus diameters, *d,* ranging from 10 up to 25 μm, prepared using the Taylor-Ulitovsky technique described elsewhere [13,14,15,16,20]. 

The electrical impedance *Z* of a magnetic conductor is expressed by [7,8]:

(1) $$Z=R_{dc}krJ_{0}(kr)/2J_{1}(kr)$$

where *R_dc_* is the *DC* electrical resistance, *k =* (1 *+ j*)/*δ*, where *J_0_* and *J_1_* are the Bessel functions, *r* is the wire’s radius and *δ* the penetration depth given by:

δ = (πσμ_ϕ_f)^−1/2^ (2)

where *σ* is the electrical conductivity, μ_ϕ_ is the circular magnetic permeability and *f* is the frequency of the current along the sample. 

For the impedance *Z* evaluation we used a micro-strip sample holder previously described elsewhere [44,45]. The glass layer at the wire ends was removed mechanically to allow making the electrical contacts. The contact resistance (tenth of ohm) was neglected as it is small (about two order lower) as compared to the wire resistance (about 20–60 ohms). Z*-*values have been measured using a vector network analyzer from the reflection coefficient, *S*_11*,*_ using expression [44]:

*Z =Z*_0_(1 *+S*_11_)/(1 *−S*_11_) (3)

where *Z*_0_ = 50 ohm is the characteristic impedance of the coaxial line. 

From Z-values obtained for different magnetic field *H* values we evaluated the magnetic field dependencies of the GMI ratio *ΔZ/Z* defined as described elsewhere [7,8,9,10]:

*ΔZ/Z* = [*Z*(*H*) − *Z*(*H_max_*)]/*Z*(*H_max_*) (4)

where *H_max_* is the maximum applied DC magnetic field.

The magnetic field H has been produced using a sufficiently long solenoid allowing to create a homogeneous magnetic field *H*.

Hysteresis loops have been measured using the fluxmetric method previously described [13,14,46]. The samples of 5 cm in length have been placed inside the single layered pick-up coil. The magnetic field has been created by 15 cm long solenoid. For this geometry the influence of the demagnetizing field and hence shape magnetic anisotropy contribution are negligible. For better comparison of magnetic wires of different compositions and post-processed at different conditions we represent the hysteresis loops as dependence of normalized magnetization *M/M_0_* on magnetic field *H* where *M* is the magnetic moment at given magnetic field and *M_0_* is the magnetic moment of the sample at the maximum magnetic field amplitude *H_m_*. 

The DW propagation has been evaluated using modified Sixtus-Tonks technique described elsewhere [13]. In contrast to the classical Sixtus-Tonks experiments [17], we used three pick-up coils for estimation of the DW velocity [13,35,36,37,38]. In the experiment 10 cm long microwire is placed coaxially inside of the long solenoid applying rectangular shaped voltage. One end of the wire is placed outside the solenoid allowing to activate DW propagation always from the opposite wire end.

The DW velocity can be estimated [13,32,35,36] as:

(5) $$v=\frac{l}{\Delta t}$$

where *l* is the distance between pick-up coils and *Δt* is the time difference between the maxima in the induced *emf*. 

We considered only the linear region of *v*(*H*) that corresponds to viscous DW propagation regime. The origin of deviations from linear *v*(*H*) dependencies at high-field region has been discussed elsewhere [13,16]. Proposed modification with 3 pick–up coils allows to avoid the contribution from multiple DW nucleation at defects [13].

Prepared microwires have been studied in as-prepared state and after annealing. Conventional and stress annealing processes have been performed in a conventional furnace. During the stress-annealing, a mechanical load has been attached to one end of the microwire and axially placed via the furnace nozzle. Such mechanical load allowed to apply tensile stress during the annealing, *σ_a_*, up to 500 MPa. All the annealing processes have been performed at temperatures below the crystallization, observed at above 450–500 °C.

## 3. Results and Discussion

### 3.1. Optimization of Magnetic Softness and GMI Effect in Magnetic Microwires

As discussed elsewhere [7,8,9,10,11], the main parameter that affects the magnitude and the magnetic field dependence of the GMI effect (including off-diagonal components) of amorphous materials is the magnetic anisotropy. Consequently, tailoring the magnetoelastic anisotropy of amorphous microwires, either through the control of the internal stresses or the magnetostriction coefficient or induced magnetic anisotropy by specially designed post-processing are the main route to optimize the GMI effect in amorphous microwires [9,15,18]. 

The primary parameter that allows to optimize the GMI effect is the minimization of the magnetostriction coefficient, *λ_s_*, either by selection of the appropriate chemical composition of the metallic alloys (usually in Co_x_Fe_1 − x_, Ni_x_Fe_1 − x_ or Co_x_Mn_1 − x_ systems for 0 ≤ x ≤ 1) [9,47,48] or alternatively by the devitrification of the amorphous precursor [49].

In the alloys based on Co_x_Fe_1 − x_ (0 ≤ x ≤ 1) binary system, the *λ_s_* values can be changed from high and positive *λ_s_* values (*λ_s_* ≈ 35 − 40 × 10^−6^) for the Fe-rich compositions (nearly zero x-values) to negative λ*_s_* values (typically *λ_s_* ≈ −5 × 10^−6^) for Co-rich alloy (x = 1) [47,48]. Similarly, *λ_s_* values can be tuned in Ni_x_Fe_1 − x_ alloys: *λ*_s_ values decrease with increasing of Ni-content is reported elsewhere [47,48,49]. However, in Fe-Ni based amorphous alloys, Ni doping originates considerable saturation magnetization and Curie temperature decrease and therefore vanishing *λ_s_* values correspond to almost paramagnetic ordering for ambient temperature [49]. 

**Figure 1:**
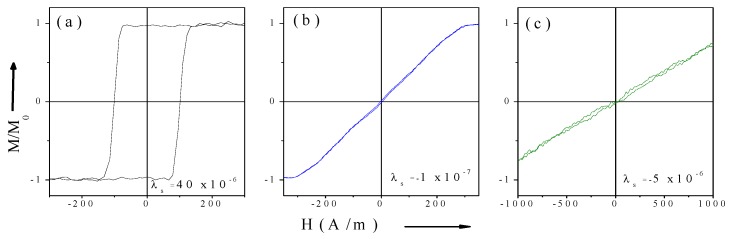
Hysteresis loops of magnetic microwires Fe_75_B_9_Si_12_C_4_ with positive (**a**) Co_67.1_Fe_3.8_Ni_1.4_Si_14.5_B_11.5_Mo_1.7_ with nearly-zero; (**b**) and Co_77.5_Si_15_B_7.5_ with negative (**c**) *λ_s_* values.

One of the examples illustrating the influence of the *λ_s_* values and sign on hysteresis loops of magnetic microwires is shown in Figure 1. 

The other important parameter that affects the magnetic softness and hence the GMI effect is the value and distribution of the internal stresses within the metallic nucleus. Such stresses are originated by rapid solidification of metallic alloy itself as well as by the different thermal expansion coefficients of the metallic alloy and glass coating [50,51,52,53,54].

**Figure 2:**
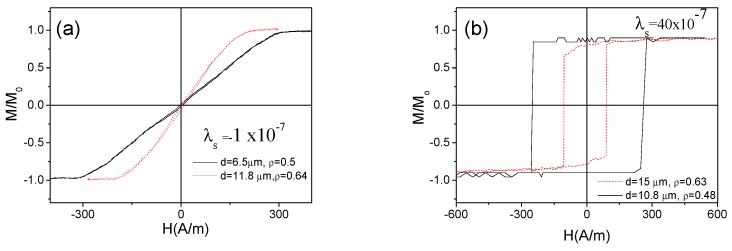
Hysteresis loops of as-prepared Co_67_Fe_3.85_Ni_1.45_B_11.5_Si_14.5_Mo_1.7_ (**a**) and Fe_75_B_9_Si_12_C_4_ (**b**) microwires with different *ρ* ratios.

The common viewpoint on internal stresses distribution [52,53,54] confirmed by a number of indirect experiments (chemical etching of glass-coating, measurements of microwires with different glass-coating thickness, effect of annealing on hysteresis loops) [55,56] is that the largest internal stresses within almost the whole metallic nucleus volume present the axial orientation. Furthermore, the internal stress values *σ_i_* increase with increasing of the glass-coating thickness [50,52], i.e., linked with the ratio *ρ* between the metallic nucleus diameter *d* and the total microwire diameter, *D* (*ρ =* *d/D*) [50,54]. Consequently, microwires with even the same composition and hence the same *λ_s_* values can present different magnetic properties. As an example, influence of the internal stresses controlled through the *ρ*–ratio for both families of microwires (Co-rich and Fe-rich) is shown in Figure 2.

It is worth mentioning that the glass-coating thickness (with lower thermal conductivity) also affects the thermal exchange conditions between the metallic nucleus and the ambient [57] and hence the quenching rate and therefore above presented description can be considered only for a first approximation.

As can be seen from Figure 1b and Figure 2a, as-prepared Co-rich microwires present typical quasi-linear hysteresis loops with very low coercivity, *H_c_* ≈ 5 A/m. 

**Figure 3:**
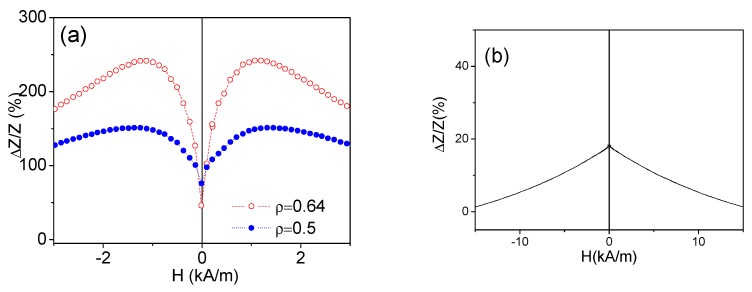
*ΔZ/Z*(*H*) dependencies of as-prepared Co_67_Fe_3.85_Ni_1.45_B_11.5_ Si_14.5_Mo_1.7_ with different *ρ*–ratios (**a**) and for as-prepared Fe_75_B_9_Si_12_C_4_ microwires; (**b**) measured at 500 MHz.

Consequently, both Co-rich microwires present high GMI ratio (see Figure 3a). On the other hand, an order of magnitude lower GMI ratio is observed in as-prepared Fe-rich microwire presenting rectangular hysteresis loop (see Figure 3b).

One more important parameter for GMI ratio optimization is the frequency. Sufficiently enough frequency *f* is one of the conditions for GMI effect observation: *f* must be high enough in order to ensure that the skin depth is below the sample radius. 

**Figure 4:**
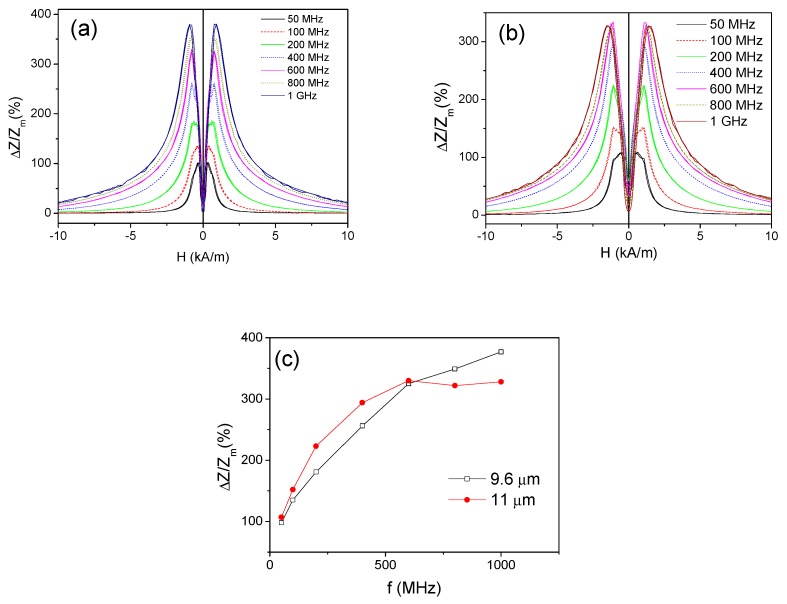
*ΔZ/Z*(*H*) dependencies measured in as-prepared Fe_3.6_Co_71.3_Ni_0.2_B_13.4_Si_10.4_C_0.9_Mo_0.2_ with d = 9.6 μm (**a**) and 11 μm (**b**) microwires and *ΔZ/Z_m_*(*f*) dependence for both microwires (**c**).

*ΔZ/Z*(*H*) dependencies measured at different frequencies in as-prepared Fe_3.6_Co_71.3_Ni_0.2_B_13.4_Si_10.4_C_0.9_Mo_0.2_ (*d =* 9.6 μm and 11 μm) microwires are shown in Figure 4a,b respectively. For comparison of the GMI effect in both samples we plotted frequency dependence of a maximum GMI ratio, *ΔZ/Z_m_*_,_ defined as a maximum *ΔZ/Z* obtained at a given frequency. Both samples have high GMI ratio, however, for the frequency range below 600 MHz the microwire with *d =* 11 μm presents superior *ΔZ/Z_m_* values (Figure 4c). However, for *f* > 600 MHz the thinner sample (*d =* 9.6 μm) presents higher *ΔZ/Z_m_* values.

Observed difference in *ΔZ/Z_m_*(*f*) dependencies for the microwires of the same composition must be related to the relationship between the wire diameter and the optimal frequency for the GMI performance: for achievement of the significant GMI effect, the penetration skin depth must be below the wire radius [58]. Consequently, the diameter reduction must be associated with rising of the optimal GMI frequency range [58]. 

It is worth mentioning that this approach has some limitations because the *ΔZ/Z_m_*(*f*) dependencies can be affected by the interfacial layer between the metallic nucleus and the glass coating observed in glass-coated microwires [59]. Indeed, different magnetic anisotropy and therefore different magnetic field dependence of the GMI ratio is expected in the interfacial layer with chemical composition different from the inner part of the metallic nucleus. 

Consequently, a number of various factors must be considered for tuning the magnetic properties in order to meet the requirements of any industrial applications. Thus, thermal treatment in some case can considerably affect magnetic properties of magnetic microwires prepared by Taylor-Ulitovsky method [39,40]. 

The other factor that must be addressed from the viewpoint of application is the cost of magnetic microwires. In this regard, Co belongs to critical raw materials [60]. Consequently, the cost of Co-rich microwires and insecure Co supplies could be potentially an obstacle for massive applications. Therefore, less expensive Fe-based microwires are preferable for large scale applications. 

In this case the nanocrystallization of Fe-rich microwires can be a useful route for improvement of magnetic softness [61,62,63]. However, amorphous materials, as a rule, present superior mechanical properties [64,65,66]. 

Therefore, search of routes allowing optimization of functional magnetic properties of amorphous microwires becomes essentially important for applications.

Consequently, below we will present our last results on optimization of magnetic softness, GMI effect and domain wall dynamics of magnetic microwires.

### 3.2. Tailoring of Magnetic Properties and Gmi Effect in Co-Rich Amorphous Microwires

One of the most common methods allowing stress relaxation is annealing. Recently, a few attempts to tailor magnetic properties and GMI effect of Co and Fe-rich microwires have been reported [38,39,40,67,68,69,70]. However, in most of the cases a magnetic hardening and deterioration of the GMI effect has been observed after conventional furnace annealing [38,39,40,67,68,69,70]. Considering that for each Co-rich composition the behavior can be different, we fixed one of the typical Co-rich compositions (Fe_3.6_Co_69.2_Ni_1_B_12.5_Si_11_Mo_1.5_C_1.2_, d = 22.8 μm, D = 23.2 μm). Below we present a systematic study of the effect of annealing conditions on hysteresis loops and GMI effect of the given microwire.

**Figure 5:**
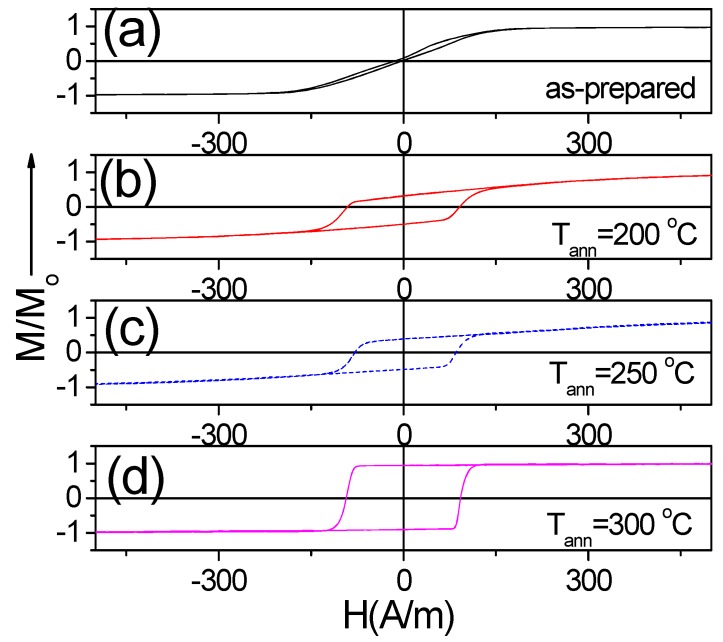
Hysteresis loops of as-prepared (**a**) and annealed at 200 °C (**b**), 250 °C (**c**) and 300 °C (**d**) Fe_3.6_Co_69.2_Ni_1_B_12.5_Si_11_Mo_1.5_C_1_ microwires.

Similarly, to other Co-rich microwires with vanishing *λ_s_* values Fe_3.6_Co_69.2_Ni_1_B_12.5_Si_11_Mo_1.5_C_1.2_ microwire presents drastic magnetic hardening: an order of magnitude coercivity rising from 5 A/m up to 90 A/m (see Figure 5). Similar magnetic hardening of Co-rich microwires after annealing is recently explained considering either magnetostriction coefficient variation and even sign change upon annealing [30,31] or stress relaxation and related domain structure modification consisting of growth of inner axially magnetized domain [23]. Therefore, conventional furnace cannot be considered as the optimal post-processing for improvement of magnetic softness at least for Co-rich microwires.

**Figure 6:**
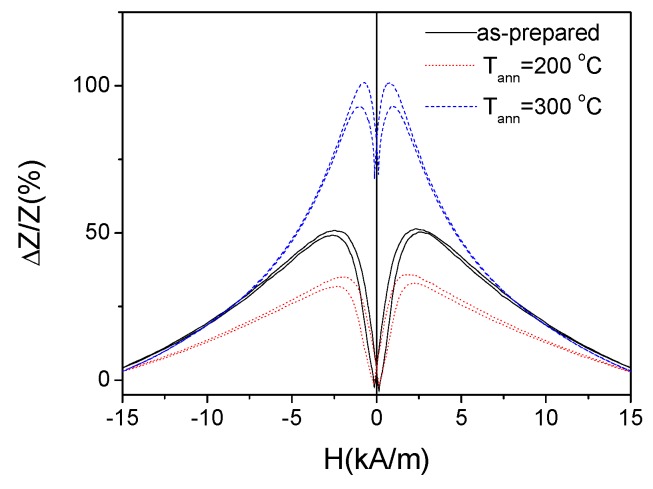
*ΔZ/Z* (*H*) dependencies of as-prepared and annealed Fe_3.6_Co_69.2_Ni_1_B_12.5_Si_11_Mo_1.5_C_1.2_ microwires measured at 500 MHz.

Therefore, as expected we observed deterioration of the GMI effect in Fe_3.6_Co_69.2_Ni_1_B_12.5_Si_11_Mo_1.5_C_1_ microwires upon annealing at 200 °C (see Figure 6). However, surprisingly GMI ratio of Fe_3.6_Co_69.2_Ni_1_B_12.5_Si_11_Mo_1.5_C_1_ microwires annealed at 300 °C is considerably higher (see Figure 6). 

**Figure 7:**
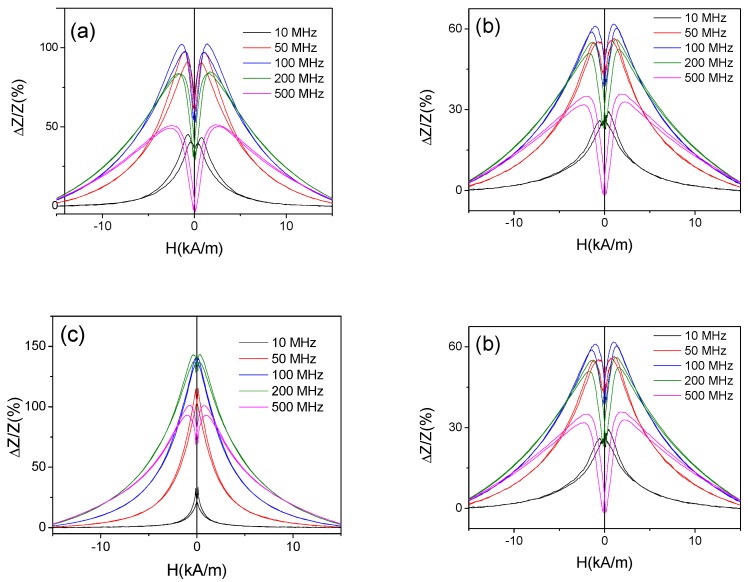
*ΔZ/Z* (*H*) dependencies of as-prepared (**a**) and annealed at 200 °C (**b**), 300 °C (**c**) and 325 °C (**d**) Fe_3.6_Co_69.2_Ni_1_B_12.5_Si_11_Mo_1.5_C_1.2_ microwires.

More detailed studies allow to observe a few more interesting dependencies: for the annealed Fe_3.6_Co_69.2_Ni_1_B_12.5_Si_11_Mo_1.5_C_1_ microwires, presenting almost perfect rectangular hysteresis loop (i.e., at T_ann_ = 300 °C and 325 °C) the GMI ratio is higher than that of as-prepared sample (see Figure 7c,d).

**Figure 8:**
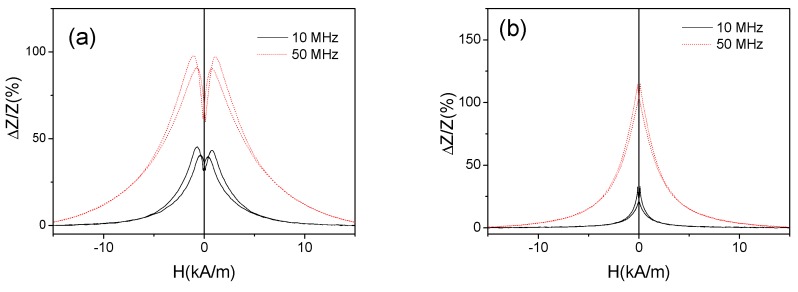
*ΔZ/Z*(*H*) dependencies of as-prepared (**a**) and annealed at 300 °C (**b**) Fe_3.6_Co_69.2_Ni_1_B_12.5_Si_11_Mo_1.5_C_1.2_ microwires.

On the other hand, the shape of *ΔZ/Z*(*H*) dependencies measured at low *f*-values (10–100 MHz) is considerably affected by the annealing: a double-maximum dependence typical for wires with circumferential magnetic anisotropy is observed for as-prepared sample, while for annealed sample *ΔZ/Z*(*H*) dependence has a shape of decay from *H* = 0 (see Figure 8). However, increasing the frequency the double maximum *ΔZ/Z*(*H*) dependencies can be observed even in annealed samples (see Figure 7a–d). 

**Figure 9:**
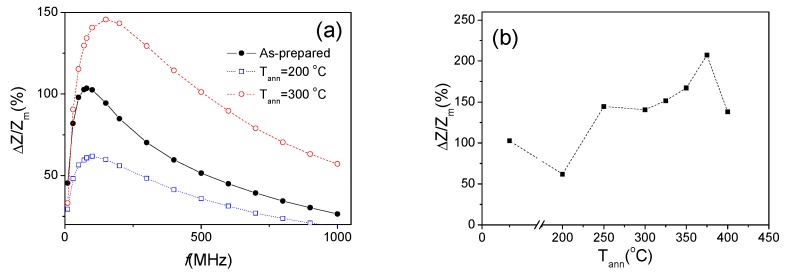
*ΔZ/Z_m_*(*f*) dependencies for as-prepared and annealed for 60 min at 200 °C and 300 °C Fe_3.6_Co_69.2_Ni_1_B_12.5_Si_11_Mo_1.5_C_1.2_ microwires (**a**) and *ΔZ/Z_m_(T_ann_)* dependence for Fe_3.6_Co_69.2_Ni_1_B_12.5_Si_11_Mo_1.5_C_1.2_ microwires measured at 100 MHz (**b**).

These experimental results are resumed in Figure 9, where dependence of maximum GMI ratio, *ΔZ/Z_m_* (defined as a maximum at a given frequency on *ΔZ/Z*(*H*) dependence for each sample) and *ΔZ/Z_m_* (T_ann_) dependencies are plotted. From these dependencies it can be deduced that from the viewpoint of the GMI ratio optimization the conventional furnace annealing can be suitable at certain annealing conditions. Additionally, observation of double-maximum *ΔZ/Z*(*H*) dependencies at higher frequencies (above 200 MHz) can be attributed to existence of thin outer shell with transverse magnetic anisotropy. In this case bulk hysteresis loop reflects only the overall magnetic anisotropy modification induced by annealing. However, for GMI effect the magnetic anisotropy in thin surface layer (with thickness comparable to the skin depth at a given frequency) is more relevant.

As recently reported [67,68,69,70], stress annealing is one of the most effective methods allowing improvement of magnetic softness and GMI ratio of magnetic microwires. Therefore, we performed stress-annealing of Co-rich microwires.

**Figure 10:**
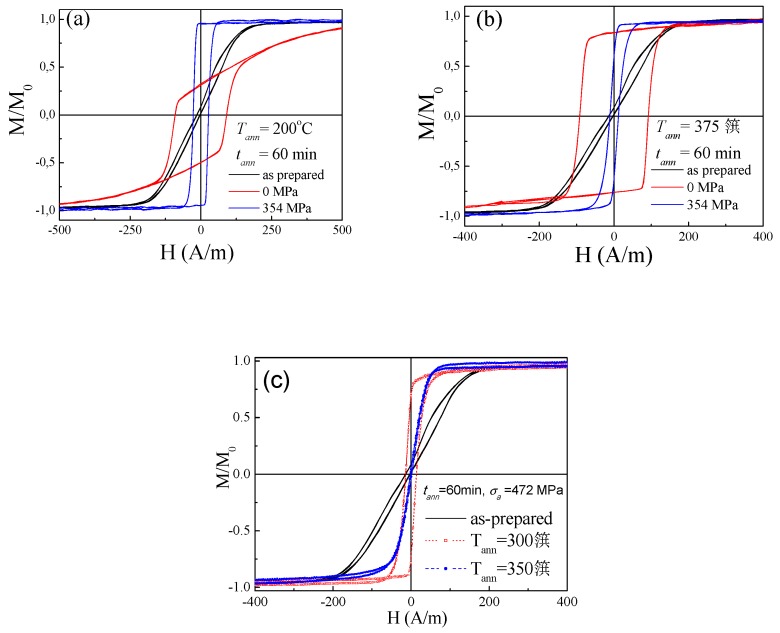
Hysteresis loops of Fe_3.6_Co_69.2_Ni_1_B_12.5_Si_11_Mo_1.5_C_1.2_ microwires as-prepared, annealed and stress-annealed at *T_ann_* = 200 °C (**a**) and *T_ann_* =375 °C (**b**) and at *t_ann_* = 60 min, *σ_a_* = 472 MPa for *T_ann_* = 300 °C and 350 °C (**c**).

The influence of the annealing parameters (*T_ann_*, *t_ann,_* *σ_a_*) on hysteresis loop of Fe_3.6_Co_69.2_Ni_1_B_12.5_Si_11_Mo_1.5_C_1.2_ microwire subjected to stress-annealing is illustrated by Figure 10. From observed evolution of the hysteresis loop we can deduce that, as-compared to conventional annealing, stress-annealing allows coercivity, *H_c_*, reduction and remanent magnetization, *M_r_/M_0_*, increase (Figure 10a). However, observed changes are affected by annealing conditions, i.e., *T_ann_*, *t_ann_,* *σ_a_*. Thus, at high enough *T_ann_* or *σ_a_* the rectangular hysteresis loop observed in stress-annealed Fe_3.6_Co_69.2_Ni_1_B_12.5_Si_11_Mo_1.5_C_1.2_ microwires becomes again linear, i.e., similar to those of as-prepared Co-rich microwires (see Figure 10c). On the other hand, the microwires annealed without stress present rectangular hysteresis loops for all *T_ann_* (see Figure 10b,c). Therefore, we can deduce that the conventional annealing (without stress) allows internal stresses relaxation. Consequently, observed increase of *H_c_* and *M_r_/M_0_* upon annealing have been explained considering modification in the magnetostriction coefficient and growing of the inner single axially magnetized domain [39,40,70]. However, for explanation of the influence of stress-annealing we can consider stress-annealing induced transverse magnetic anisotropy [70,71]. This stress-annealing induced transverse magnetic anisotropy becomes more relevant with increase of *T_ann_*, *t_ann_*, *σ_a_* [70,71]. Therefore, stress-annealed Fe_3.6_Co_69.2_Ni_1_B_12.5_Si_11_Mo_1.5_C_1.2_ microwires (annealed at high enough *T_ann_*, *t_ann_,* *σ_a_*) present lower *M_r_/M_0_*-values [71].

**Figure 11:**
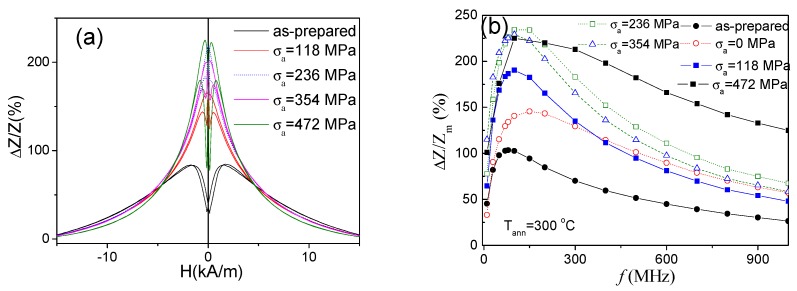
*ΔZ/Z*(*H*) dependences of as-prepared and stress-annealed at *T_ann_* = 300 °C samples at different *σ_a_* measured at 200 MHz (**a**) and *ΔZ/Z_m_*(*f*) dependencies for the same samples (**b**).

As regarding the GMI effect, all stress-annealed Co-rich microwires present a remarkable GMI ratio improvement (see comparison in Figure 11a): for *f* = 200 MHz more than double GMI ratio improvement is observed almost for all stress-annealed samples. As can be appreciated from the *ΔZ/Z_m_*(*f*) dependencies shown in Figure 11b. This *ΔZ/Z_m_* improvement is observed for all frequency range (up to 1 GHz). It is interesting that the optimum frequency for as-prepared sample is about 100 MHz, however, for stress-annealed samples the optimal frequency shifts to about 150 MHz. The highest *ΔZ/Z_m_* ratio (of about 230%) is observed for the sample annealed at *σ_a_* =236 MPa. However, the sample annealed under *σ_a_* = 472 MPa presents the highest *ΔZ/Z_m_* in the frequency range *f* ≥ 200 MHz (see Figure 11b).

Consequently, we can resume that the stress-induced anisotropy allows tuning of both the GMI ratio value and the *ΔZ/Z_m_*(*f*) dependencies.

Most of hysteresis loops of annealed and stress-annealed Fe_3.6_Co_69.2_Ni_1_B_12.5_Si_11_Mo_1.5_C_1.2_ microwires present rectangular shape. Therefore, it is expected that the remagnetization process runs by single domain wall propagation as described elsewhere [11,12,13,14].

**Figure 12:**
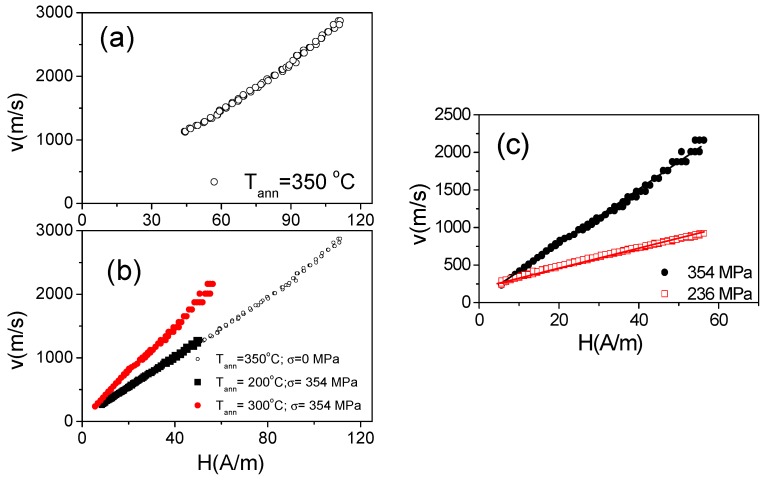
*v*(*H*) dependencies measured for the Fe_3.6_Co_69.2_Ni_1_B_12.5_Si_11_Mo_1.5_C_1.2_ samples annealed at *T_ann_* = 350 °C (**a**) for the sample annealed at *σ_a_* = 0 MPa and *σ_a_* = 354 MPa for different *T_ann_* (**b**) and at *T_ann_* = 350 °C for different applied stress (**c**).

Experimental results on DW dynamics of studied sample are presented in Figure 12. Indeed, annealed sample presents quite fast DW propagation: for the sample annealed at *T_ann_* = 350 °C the DW velocity *v* up to 3000 m/s can be observed (Figure 12a). The DW dynamics is affected by *T_ann_* (Figure 12b) and *σ_a_* (Figure 12c). One of the features of the DW dynamics in stress-annealed Fe_3.6_Co_69.2_Ni_1_B_12.5_Si_11_Mo_1.5_C_1.2_ microwires is that the magnetic field range at which the single DW propagation can be observed is shifted to low –field region (see Figure 12b) as compared to annealed (for *σ_a_* = 0 MPa) samples. 

Furthermore, the DW dynamics in all annealed samples can be described by linear *v*(*H*) dependencies previously attributed to a viscous regime [35,36,37,38]:

*ν* = *S*(*H* − *H_0_*) (6)

where *S* is the DW mobility and *H_0_* is the critical propagation field, below which the DW propagation cannot be observed.

As observed from Figure 12, *v* and *S*-values increase rising the *T_ann_ (*Figure 12b) and *σ_a_* (Figure 12c).

As evidenced from observed dependencies both furnace annealing and especially stress-annealing are promising methods allowing optimization of functional magnetic properties, such as GMI ratio, coercivity and DW velocity and mobility.

### 3.3. Tailoring of Gmi Effect and Domain Wall Dynamics in Fe-Rich Microwires

As mentioned above, the magnetic softness of Fe-rich microwires is generally limited by high enough magnetostriction coefficient, *λ_s_*, values. One of the routes allowing considerable reduction of *λ_s_* is the nanocrystallization, usually observed either in Finemet-type [49,61,62,63] or Hitperm-like magnetic wires [72]. However, generally nanocrystalline materials present poor mechanical properties [66]. Therefore, applications of nanocrystalline magnetic microwires are sufficiently restricted by poorer mechanical properties.

The alternative method allowing magnetic softening of magnetic microwires lies in the selection of appropriate post-processing aimed to reduce the magnetoelastic anisotropy i.e., by redistribution of the internal stresses [67,68,69,70].

Below we will present some last experimental results on tuning of GMI effect and DW dynamics of an amorphous Fe-rich microwire with quite typical composition (Fe_75_B_9_Si_12_C_4_; *d* = 15,2 μm; *D* = 17,2 μm) with high and positive magnetostriction coefficient (*λ_s_* ≈ 38 × 10^−6^) [48,73] by post-processing.

**Figure 13:**
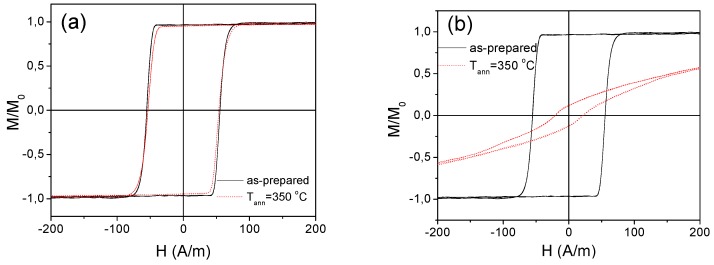
Hysteresis loops of as-prepared and annealed at *T_ann_* = 350 °C for *σ_a_* = 0 MPa Fe_75_B_9_Si_12_C_4_ sample (**a**) and annealed at the same *T_ann_* for *σ_a_* = 190 MPa Fe_75_B_9_Si_12_C_4_ sample (**b**).

As can be appreciated from Figure 13, as-prepared and annealed (without stress) Fe_75_B_9_Si_12_C_4_ samples present rectangular hysteresis loops, as reported elsewhere for microwires with positive *λ_s_* values [13,35,36,37,38]. Slight *H_c_* decreasing after annealing is observed (see Figure 13a for *T_ann_* = 350 °C).

However, rather different hysteresis loop is observed in the stress-annealed (at the same *T_ann_*) Fe_75_B_9_Si_12_C_4_ sample (see Figure 13b): The hysteresis loop becomes almost linear (see Figure 13a,b).

**Figure 14:**
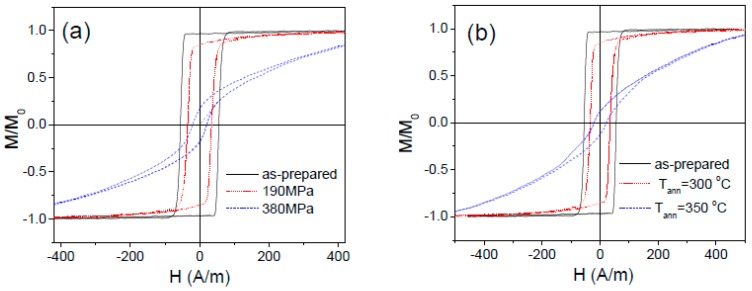
Effect of tensile stress applied during the annealing at 300 °C (*t_ann_* = 60 min) (**a**) and annealing temperature at fixed *σ_a_* = 190 MPa; (**b**) on hysteresis loops of Fe_75_B_9_Si_12_C_4_ microwire.

Observed stress-annealing induced magnetic anisotropy depends on a few parameters: *T_ann_*, *σ_a_* (see Figure 14a,b). Increasing the annealing temperature and stress applied during the annealing a decrease in remnant magnetization, *M_r_/M_o_*, and *H_c_* and increase in magnetic anisotropy field, *H_k_,* is observed (see Figure 14a,b). Consequently, Fe_75_B_9_Si_12_C_4_ microwires annealed at high enough *T_ann_* or *σ_a_* present strong transverse magnetic anisotropy characterized by high *H_k_* values (see Figure 14a,b).

**Figure 15:**
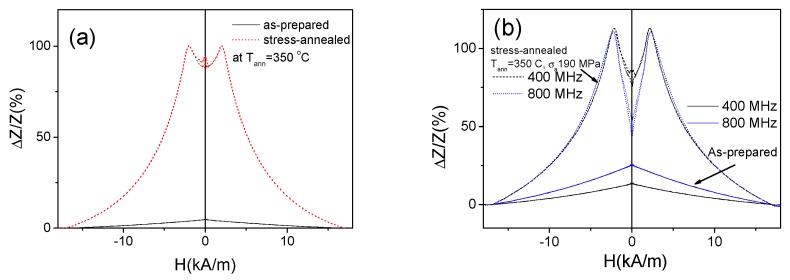
*ΔZ/Z*(*H*) dependencies observed in as-prepared and stress-annealed at *T_ann_* = 350 °C for *σ*_a_ = 190 MPa Fe_75_B_9_Si_12_C_4_ microwires (*f* = 200 MHz) (**a**) and the same dependencies measured at 400 and 800 MHz (**b**).

Observed considerable magnetic softening evidenced from Figure 13 and Figure 14 allows a remarkable GMI ratio improvement (more than an order of magnitude) (see Figure 15). This GMI effect must be attributed to stress-annealing induced transversal magnetic anisotropy and related improvement of circumferential magnetic permeability. 

Rather different magnetic anisotropy of as-prepared and stress-annealed Fe_75_B_9_Si_12_C_4_ microwires can be deduced from *ΔZ/Z*(*H*) dependencies of both samples (see Figure 15a,b). As observed in Figure 15a,b, single peak *ΔZ/Z*(*H*) dependence with *ΔZ/Z* maximum at *H* = 0 is observed for all frequencies. Such kind of *ΔZ/Z*(*H*) dependence with decay from *H* = 0 is predicted for axial magnetic anisotropy (also evidenced from the hysteresis loops) [28]. 

**Figure 16:**
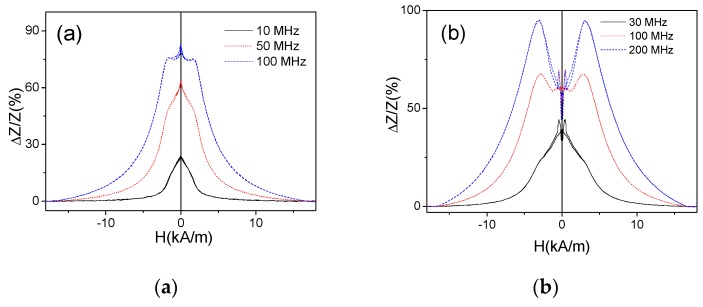
*ΔZ/Z*(*H*) dependencies observed in stress-annealed at *T_ann_* = 350 °C (for 60 min and *σ_a_* = 190 MPa) (**a**) and at T_ann_ = 300 °C (for 60 min and *σ_a_* = 900 MPa) (**b**) Fe_75_B_9_Si_12_C_4_ microwires.

The stress-annealed (*T_ann_* = 350 °C for *σ*_a_ = 190 MPa) Fe_75_B_9_Si_12_C_4_ microwire presents rather unusual and irregular *ΔZ/Z*(*H*) dependence for intermediate frequency range (100–400 MHz) (see Figure 15a,b). However, rising the frequency a double-peak *ΔZ/Z*(*H*) dependence of stress-annealed Fe_75_B_9_Si_12_C_4_ microwire is observed (see Figure 15b). On the other hand, for low frequencies (*f* ≤ 50 MHz) the single peak *ΔZ/Z*(*H*) dependence of stress-annealed Fe_75_B_9_Si_12_C_4_ microwire is observed (see Figure 16a). Similar behavior is observed for the Fe_75_B_9_Si_12_C_4_ microwires stress-annealed at different conditions: single-peak dependence for low *f* region, double peak for high enough frequencies and irregular *ΔZ/Z*(*H*) dependence for intermediate *f* –values (see Figure 16).

Observed irregular *ΔZ/Z*(*H*) dependence recently has been attributed to as the superposition of the double-peak *ΔZ/Z*(*H*) dependence typical for transverse magnetic anisotropy and single-peak dependence reported for axial magnetic anisotropy [74]. Evolution of *ΔZ/Z*(*H*) dependencies rising the frequency can be explained considering frequency dependence of the penetration depth, *δ*, given by Equation 2. Indeed, as followed from Equation 2 and reported previously elsewhere [75], a decrease in *δ* with *f* increasing is expected. On the other hand, it is commonly considered that the domain structure of magnetic wires consists of inner axially magnetized core and outer domain shell [76,77,78]. The evolution of such domain structure upon annealing can be evaluated considering the relationship of the squareness ratio, *M_r_/M_o_* with the radius of the inner axially magnetized core, *R_c_*, given as [76]:

*R_c_* = *R*(*M_r_/M_o_*)^l/2^ (7)

where *R* is the metallic nucleus radius. 

A remarkable *M_r_/M_o_* decrease is observed upon stress annealing (see Figure 13 and Figure 14).

Therefore, a contribution of both inner axially magnetized core and outer domain shell with circumferential magnetic anisotropy is expected for low frequencies. However, at high enough frequencies *δ* becomes smaller and therefore, the only contribution of the outer domain shell can be assumed.

**Figure 17:**
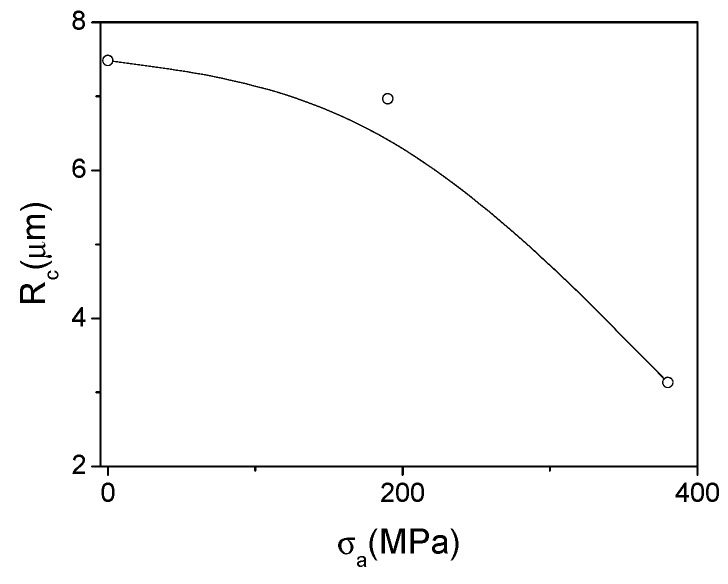
Effect of the stress applied during the annealing at *T_ann_* = 300 °C on *R_c_* values of Fe_75_B_9_Si_12_C_4_ microwire. The line is for the eyes only.

From the hysteresis loops presented in Figure 14a we obtained the *M_r_/M_o_*-values and evaluated the *R_c_* dependence on *σ_a_*-values (at *T_ann_* = 300 °C, see Figure 17). As observed from Figure 17, *R_c_*-values progressively decrease with *σ_a_*-values increasing (at *T_ann_* = 300 °C). Consequently, from *R_c_*(σ_a_) dependence we can assume an increase of the outer domain shell volume with transverse magnetization orientation in expense of a decreasing in the inner axially magnetized core volume. 

**Figure 18:**
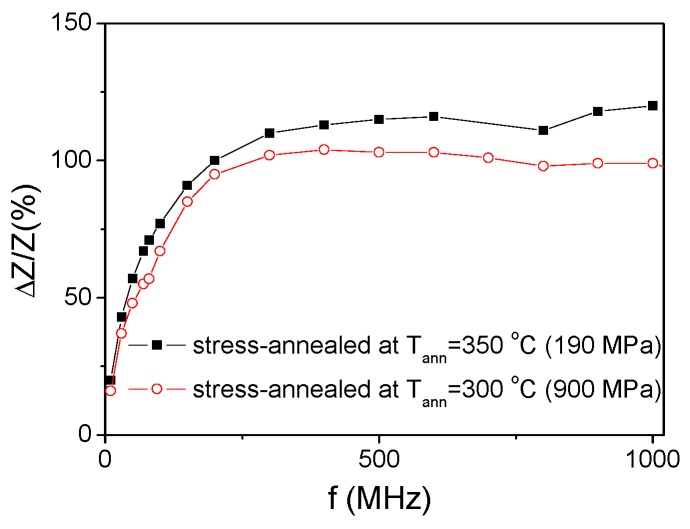
Frequency dependence of maximum GMI ratio for stress-annealed (*T_ann_* = 350 °C, *σ_a_* = 190 MPa and *T_ann_* =300 °C, *σ_a_* = 900 MPa) Fe_75_B_9_Si_12_C_4_ microwires.

One more relevant feature of stress-annealed Fe_75_B_9_Si_12_C_4_ microwires is elevated *ΔZ/Z_m_* values observed in a wide frequency range evidenced from the *ΔZ/Z_m_*(*f*) dependencies shown in Figure 18. It is worth mentioning that *ΔZ/Z_m_* ≥ 100% are observed for the frequency between 200 MHz and 1 GHz (see Figure 18).

Generally, Fe-rich microwires are commonly recognized as a material suitable for experimental studies of single DW propagation and potentially interesting for related applications [13,16,32,33,34,35,36,37,38]. These features are closely linked to spontaneous magnetic bistability exhibited by as-prepared Fe-rich microwires [13,14]. Consequently, single and fast DW propagation of Fe-rich microwires became a subject of intensive research [13,35,36,37,38]. 

**Figure 19:**
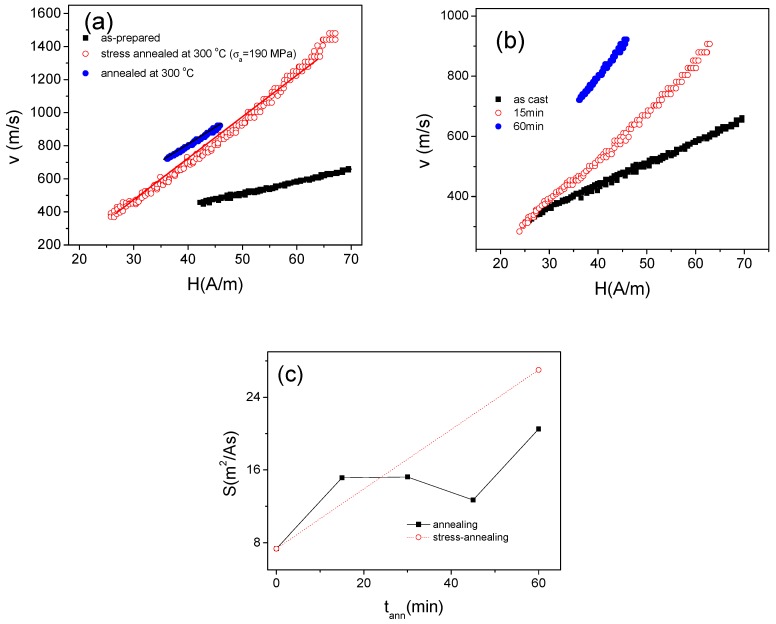
*v*(*H*) dependencies of as-prepared, annealed (*T_ann_* = 300 °C) and stress- annealed at *T_ann_* = 300 °C for *σ_a_* =190 MPa (**a**) for as-prepared and annealed at *T_ann_* = 300 °C for different *t_ann_* (**b**) and effect of annealing conditions on *S*-value (**c**) of Fe_75_B_9_Si_12_C_4_ microwires. The lines are just for eyes.

Previously it was shown that the DW dynamics can be considerably improved by minimization of the magnetoelastic anisotropy [13,35,36,37,38]. One of the most effective methods is selection of the appropriate chemical composition of metallic nucleus with low magnetostriction coefficient [13,35,36]. However, even for fixed chemical composition the DW dynamics can be considerably improved by annealing allowing internal stresses relaxation [40]. Furthermore, stress-annealing induced anisotropy can improve even more efficiently the DW dynamics [71]. The effect of annealing and stress-annealing on *v*(*H*) dependencies of Fe_75_B_9_Si_12_C_4_ microwires is resumed in Figure 19. Similarly, to previously reported results [38,40,71,79], a remarkable improvement of DW velocity, *v,* and DW mobility, *S*, is observed (see Figure 19a). It is worth noting that the DW velocity can be improved up to 1.5 km/s (see Figure 19) by stress- annealing. This dependence of DW dynamics is affected by a number of parameters, i.e., annealing time, *t_ann_*, at fixed *T_ann_* (see Figure 19b). However, in the case of stress-annealing the samples annealed long enough time, at high enough *σ_a_* or *T_ann_* do not present magnetic bistability (see Figure 13 and Figure 14). The DW mobility, *S*, rises from about *S* ≈ 7.5 m^2^/A∙s for as-prepared sample up to *S* ≈ 20 m^2^/A∙s (annealed at *T_ann_* = 300 °C) and even up to *S* ≈ 27 m^2^/A∙s (stress- annealed at *T_ann_* = 300 °C, see Figure 19c). However, for *T_ann_* = 300 °C we have experimental results only for *t_ann_* = 60 min (shown as dot line only for comparison). Even more remarkable effect of stress-annealing on *S-* values (*S* up to 40 m^2^/A∙s) is reported recently for *T_ann_* = 325 °C [71].

The interpretation of the effect of conventional annealing on DW dynamics involves the magnetoeslastic anisotropy contribution to the domain wall mobility, *S*, given by [38,80,81]:

*S* = 2*μ*_0_*Μ_s_/β* (8)

where *β* is the viscous damping coefficient, *μ_0_* is magnetic permeability of vacuum. 

Although generally three contributions have been discussed elsewhere [38,81], i.e.,

- The eddy current contribution, *β_e_*, is associated to the micro-eddy currents circulating nearby moving domain wall;
- The magnetic relaxation damping, *β_r_*, related to the Gilbert damping parameter, *α*;
- The structural relaxation contribution originated from the interaction of mobile defects with the local magnetization [81].

The most evident interpretation is related to the magnetic relaxation damping, *β_r_*, which is related to the anisotropy constant, *K* through the DW width *δw* [38]

*β_r_* ≈ 2*απ*^−1^(*K/A*)^1/2^ (9)

where *A* is the exchange interaction constant. As mentioned above, in amorphous materials the main origin of magnetic anisotropy is the magnetoelastic anisotropy, *K_me_*, given by:

*K_me_* ≈ 3/2*λ_s_σ_i_* (10)

where *λ_s_* is the magnetostriction coefficient, and *σ_i_* - the internal stress.

In the latter case the annealing influence must be related to the internal stresses relaxation [38,79,81].

Indeed, elevated internal stresses are intrinsically related to the preparation method involving rapid melt quenching of metallic nucleus surrounded by the glass coating [50,51,52,53,54].

The interpretation of the stress-annealing influence of DW dynamics in magnetic microwires is recently given considering the similarity of the stress-annealing induced magnetic anisotropy and transverse magnetic field on DW dynamics [79]. Indeed, beneficial effect of transverse magnetic field on the DW dynamics is recently reported in a few publications [82,83,84,85]. The interpretation involves the influence of the transverse magnetic field on the DW structure and the DW energy landscape. 

Aforementioned examples provide the routes for optimization of GMI effect and DW dynamics in Co-and Fe-rich microwires. 

## 4. Conclusions

We concluded that the GMI effect, magnetic softness or DW dynamics of microwires can be tailored by controlling the magnetoelastic anisotropy of as-prepared microwires either through tuning of their internal stresses, magnetostriction coefficient and hence domain structure or by appropriate thermal treatment. 

The impact of stress-annealing on magnetic properties and domain wall (DW) propagation and giant magneto-impedance (GMI) effect in Co and Fe- rich microwires is experimentally studied. Observed stress-induced anisotropy is considerably affected by annealing conditions (annealing time, temperature or stress applied during the annealing).

Considerable magnetic hardening and transformation of linear hysteresis loop with low coercivity (*H_c_* ≈ 4 A/m) into rectangular with *H_c_* ≈ 90 A/m upon annealing without stress is observed in Co-rich microwires. However, considerable MI effect improvement at certain annealing conditions is observed. Even more remarkable improvement of the GMI effect is observed in Co-rich glass-coated microwires subjected to stress annealing.

Remarkable improvement of DW mobility and GMI ratio are achieved by stress-annealing in Fe-rich microwires. Furthermore, the shape of *ΔZ/Z*(*H*) dependencies for as-prepared and stress-annealed samples present considerable difference. A remarkable effect of stress-annealing on hysteresis loops is attributed to the domain structure modification: Rising the volume of the outer domain shell with transverse magnetic anisotropy. We assumed that this outer domain shell with transverse magnetic anisotropy affects the travelling DW in a similar way as application of transversal bias magnetic field allowing enhancement the DW velocity. Accordingly, stress annealing of Fe-rich microwires allowed us to achieve the magnetic anisotropy distribution beneficial for optimization of either the GMI effect or the DW dynamics.

Consequently, versatile properties of magnetic microwires (DW dynamics, magnetic softness and GMI effect) can be optimized by appropriate postprocessing.
